# Glass and digital transformation

**DOI:** 10.1007/s40940-021-00148-8

**Published:** 2021-03-05

**Authors:** Jens Schneider, Jan Belis, Christian Louter, Jens Henrik Nielsen, Mauro Overend

**Affiliations:** 1 Institute of Structural Mechanics and Design, Technische Universität Darmstadt, Darmstadt, Germany; 2 Department of Structural Engineering and Building Materials, Ghent University, Ghent, Belgium; 3 Institute of Building Construction, Technische Universität Dresden, Dresden, Germany; 4 Department of Civil Engineering, Technical University of Denmark, Kgs. Lyngby, Denmark; 5 Faculty of Architecture and the Built Environment, Delft University of Technology, Delft, The Netherlands

A new issue is ready and the world is still trapped in the pandemic of COVID19. Many of us work from home, at least partially. Traveling, especially in an international context, is restricted. Performing research and providing education is challenging. Although we aim for *physical* *distancing* only, *social distancing* is one of the negative effects of the measures implemented to reduce the spread of the virus and its mutants.

On the other hand, a crisis like the COVID-19 pandemic, also opens new chances. For example, the digital transformation of our societies gets a real push and might help us to develop a more sustainable behaviour. Videoconferences are now fully accepted, beneficial effects of remote teaching become visible, and thus in the future we will be better able to evaluate if travels are really needed.

The digital transformation also affects our glass research – and this will definitely continue: Digital image processing is used in the paper of Dix et al. for the evaluation of anisotropy effects in pre-stressed glass; Drass et al. use semantic segmentation with deep learning for the detection of cracks at cut edges of flat glass, Hayez et al. give design rules for silicone joints in cold bent glass based on numerical simulations.

The other papers in the current issue focus on the glass itself and material combinations with glass. Pauli et al. perform experimental and numerical investigations on glass fragments to derive a material model which in the future might be used to enhance post-fracture models for laminated glass, Brokmann et al. revisit the well-known problem of subcritical crack growth as a function of the environmental conditions, Joachim et al. perform testing of a novel combination of materials in composite panels made of glass and fibre-reinforced plastics and finally Götzinger et al. show first results of a new type of glass laminates adding paper as an interlayer.

Stay healthy and keep reading our journal - especially if you are trapped at home!

